# PD-1 as a potential target in cancer therapy

**DOI:** 10.1002/cam4.106

**Published:** 2013-07-21

**Authors:** David F McDermott, Michael B Atkins

**Affiliations:** 1 Biologic Therapy Program, Beth Israel Deaconess Medical CenterBoston, Massachusetts; 2 Harvard Medical SchoolBoston, Massachusetts; 3 Georgetown-Lombardi Cancer Center, Georgetown University School of MedicineWashington, District of Columbia

**Keywords:** Cancer, immune tolerance, immunotherapy, nivolumab, programmed cell death-1 receptor, programmed cell death-1-ligand 1

## Abstract

Recently, an improved understanding of the molecular mechanisms governing the host response to tumors has led to the identification of checkpoint signaling pathways involved in limiting the anticancer immune response. One of the most critical checkpoint pathways responsible for mediating tumor-induced immune suppression is the programmed death-1 (PD-1) pathway, normally involved in promoting tolerance and preventing tissue damage in settings of chronic inflammation. Many human solid tumors express PD ligand 1 (PD-L1), and this is often associated with a worse prognosis. Tumor-infiltrating lymphocytes from patients with cancer typically express PD-1 and have impaired antitumor functionality. Proof-of-concept has come from several preclinical studies in which blockade of PD-1 or PD-L1 enhanced T-cell function and tumor cell lysis. Three monoclonal antibodies against PD-1, and one against PD-L1, have reported phase 1 data. All four agents have shown encouraging preliminary activity, and those that have been evaluated in larger patient populations appear to have encouraging safety profiles. Additional data are eagerly awaited. This review summarizes emerging clinical data and potential of PD-1 pathway–targeted antibodies in development. If subsequent investigations confirm the initial results, it is conceivable that agents blocking the PD-1/PD-L1 pathway will prove valuable additions to the growing armamentarium of targeted immunotherapeutic agents.

Next-generation immunotherapy agents that target the PD-1 checkpoint pathway are demonstrating antitumor activity and encouraging safety profiles in early clinical trials. Current and future clinical trials will provide new insights, and the evaluation of biomarkers and rational combination therapies is ongoing.

## Introduction

Multiple immunotherapeutic approaches to cancer treatment have been evaluated over the past several decades. Although the results of many of these early efforts have been disappointing, the ability to produce durable remissions of solid tumors with high-dose interleukin-2 (HD IL-2), interferon-α, and vaccines has nevertheless provided evidence of immunotherapy's potential 1–3. Recent data have provided a clearer understanding of the factors that limit an antitumor immune response, leading to the development of various agents targeting immune costimulatory and inhibitory (“checkpoint”) pathways. One of the key checkpoint molecules that mediates tumor-induced immune suppression is programmed death-1 (PD-1).

Traditional costimulation is delivered by the signaling of antigen-presenting cell (APC) CD80/86 through T-cell CD28, the so-called “second signal” required for T-cell activation. In addition to CD28, other immune costimulatory molecules include inducible costimulator 4, CD137 (also known as 4-1BB), and OX40 5. Conversely, several negative regulatory checkpoint molecules function to prevent, or “check,” overstimulation of immune responses and contribute to the maintenance of immune tolerance to self-antigens 6. These molecules include cytotoxic T-lymphocyte antigen-4 (CTLA-4) as well as the PD-1 receptor and its ligands. CTLA-4 acts as a signal dampener that acts largely within the lymph nodes to regulate the magnitude of early activation of naïve and memory T cells. PD-1, by contrast, is induced on T cells after activation in response to inflammatory signals and limits T-cell function in various peripheral tissues, largely in the context of infection or tumor progression 7. As the T-cell response builds, these negative regulatory molecules are induced, limiting the magnitude and duration of the response to prevent healthy tissue damage.

Tumors are capable of exploiting the homeostatic mechanisms regulated by these checkpoint molecules. They can overwhelm the immune system via multiple strategies, including alterations in antigen expression, interference with T-cell priming, and a spectrum of effects referred to as “immune editing,” whereby tumors manipulate their microenvironment during development to escape immune detection and eradication 8. Limiting antitumor T-cell responses via exploitation of checkpoint pathways (such as those involving CTLA-4 or PD-1) serves to prevent significant tumor destruction and leads to an equilibrium between the tumor and immune system that typically progresses to tumor escape. New immunotherapies for cancer focus on shifting the balance from a pro-tumor to an antitumor microenvironment, thus allowing the immune system to mount an effective antitumor response; consequently, negative regulatory pathways are key targets. The anti–CTLA-4 monoclonal antibody (mAb) ipilimumab improved survival in a phase 3 trial in patients with metastatic melanoma (MEL) 9 and was subsequently approved by the United States Food and Drug Administration for the treatment of patients with advanced MEL. A recent report of an early-stage trial has provided preliminary evidence of activity of ipilimumab in patients with castrate-resistant prostate cancer (CRPC) 10. The fully human anti–PD-1 mAb BMS-936558/MDX-1106/ONO-4538 (nivolumab), tested in renal cell cancer (RCC), MEL, CRPC, non–small cell lung cancer (NSCLC), and colorectal cancer (CRC), has demonstrated antitumor activity in phase 1/1b studies 11. The humanized anti–PD-1 antibody MK-3945 (lambrolizumab) has also demonstrated antitumor activity in patients with solid cancers in a phase 1 study 12. CT-011 (pidilizumab), a humanized anti–PD-1 antibody, has been evaluated in multiple hematologic malignancies, demonstrating potential clinical activity in patients with non-Hodgkin's lymphoma, chronic lymphocytic leukemia, Hodgkin's lymphoma, multiple myeloma, and acute myeloid leukemia 13. Finally, the anti–PD ligand 1 (PD-L1) mAb BMS-936559 has shown preliminary antitumor activity in various solid cancers 14. PD-1 pathway–targeted agents in development are summarized in Table 1. This review examines the role of the PD-1 negative regulatory pathway in antitumor immune responses and outlines the rationale for targeting PD-1 and PD-L1 in the treatment of patients with cancer.

**Table:** PD-1 pathway–targeted agents in development

Target	Name	Description	Sponsor	Phase
PD-1	Nivolumab	Fully human IgG4 monoclonal antibody	Bristol-Myers Squibb	3
	Lambrolizumab	Humanized IgG4 monoclonal antibody	Merck	3
	Pidilizumab	Humanized IgG1 monoclonal antibody	CureTech	2
	AMP-224	B7-DC/IgG1 fusion protein	GlaxoSmithKline/Amplimmune	1
PD-L1	BMS-936559	Fully human IgG4 monoclonal antibody	Bristol-Myers Squibb	1
	RG7446/MPDL3280A	Monoclonal antibody	Roche/Genentech	1
	MEDI4736	Monoclonal antibody	MedImmune	1

## Role of the PD-1 Pathway in the Immune Response

PD-1 (CD279), a member of the B7-CD28 family 15, is a cell surface coinhibitory receptor expressed on T cells, B cells, monocytes, and natural killer T cells, following activation 16. PD-1 has two known ligands: PD-L1 (B7-H1) 17 and PD-L2 (B7-DC) 18, which have distinct expression profiles. Both ligands are expressed on APCs, including dendritic cells (DCs); in addition, PD-L1, thought to be the principal mediator of PD-1-dependent immunosuppression 19, is expressed on some nonhematopoietic cells. Binding of PD-L1 or PD-L2 to PD-1 inhibits T-cell receptor signaling and downregulates the expression of certain antiapoptotic molecules (including B-cell lymphoma-extra large [Bcl-xL]) and proinflammatory cytokines 16. The interaction of PD-1 with its ligands also affects the cell cycle, preventing progression through G1 phase by increasing expression levels of p15 and suppressing transcription of *SKP2*, a component of the ubiquitin ligase responsible for degrading p27 20. PD-L1 itself has also been observed to serve as an antiapoptotic factor on tumor cells 21. Interestingly, PD-L1 expressed on APCs can also bind T-cell CD80, which curtails T-cell activation and cytokine production. The PD-1 pathway is an important regulator of the induction and maintenance of peripheral tolerance (and tolerance to malignant “self” cells within the tumor microenvironment) 22,23, by upholding a balance between T-cell activation and the protection of healthy tissues from immune-mediated damage.

**Figure 1:**
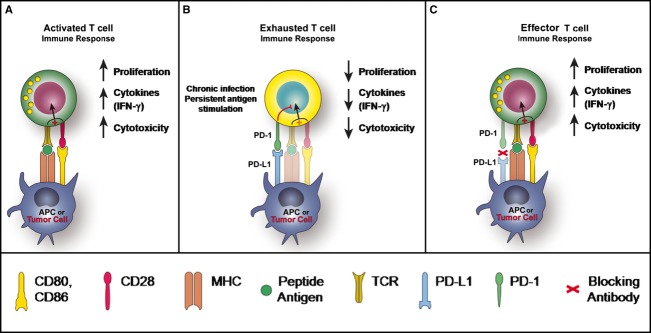
PD-1 in T-cell activation, exhaustion, and effector function. (A) T cells are activated via (1) binding of MHC plus peptide on an APC to the TCR and then (2) binding of APC CD80/86 to T-cell CD28. In patients with cancer, tumor cells can also serve as APCs. Upon T-cell activation, PD-1 expression is induced. (B) In situations of chronic infection or persistent stimulation, PD-L1 signals through T-cell PD-1 to “turn off” T cells in order to minimize damage to healthy tissue. Tumor cells can upregulate PD-L1 in order to “turn off” T cells that might destroy them. (C) Blocking the PD-1/PD-L1 signaling pathway allows T cells to maintain their effector functions. In patients with cancer, activated tumor-specific T cells can kill tumor cells and secrete cytokines that activate/recruit other immune cells to participate in the antitumor response. APC, antigen-presenting cell; IFN-γ, interferon gamma; MHC, major histocompatibility complex; PD-1, programmed death-1; PD-L1, PD ligand 1; TCR, T-cell receptor.

The critical role of the PD-1 pathway in blunting T-cell responses was first demonstrated by the various autoimmune phenotypes observed in PD-1 knockout mice 16. In addition, PD-L1 expression on nonhematopoietic cells was shown to inhibit immune-mediated tissue damage 21,24. The PD-1/PD-L1 pathway also participates in the regulation of immunity to chronic infections. Persistent T-cell activation in the setting of chronic infection produces sustained, high-level PD-1 expression and results in functionally exhausted T cells. Such T cells are unable to proliferate and destroy invading microorganisms, thus allowing infections to persist; however, this inactivity of T cells also reduces collateral immune-related damage to host cells 25. Cancers exploit multiple immunoregulatory pathways to evade elimination by infiltrating, activated tumor-specific T cells, including the production of immunosuppressive cytokines (transforming growth factor-β [TGF-β], interleukin-10 [IL-10]), the expression of immunosuppressive enzymes (indoleamine-2,3-dioxygenase), and the conversion of “normal” APCs and T cells to immunosuppressive cell populations (e.g., regulatory T cells) 26. Similarly, tumor upregulation of PD-L1 expression, commonly induced by infiltrating T-cell release of interferon-γ (IFN-γ) 27, leads to inactivation of T cells expressing PD-1, further enabling tumor cell evasion of immune destruction (Fig. 1).

## Role of the PD-1 Pathway in Cancer

Gajewski and colleagues have recently observed that certain tumors exhibit an “inflammatory” gene signature, suggesting the existence of an ongoing immune response; moreover, patients with “inflamed” tumors appear to respond better to immunotherapy. Intriguingly (and perhaps counter-intuitively), “inflamed” tumors contained higher expression levels of the immunosuppressive molecules PD-L1, FoxP3 (the transcription factor controlling regulatory T-cell [Treg] development) and indoleamine-2,3-dioxygenase (an enzyme critically involved in peripheral tolerance) 28. The PD-1 pathway, therefore, may have a key role in the interaction of tumor cells with the host immune response, and tumor cell PD-L1 expression may serve as a mechanism of adaptive immune resistance.

**Figure 2:**
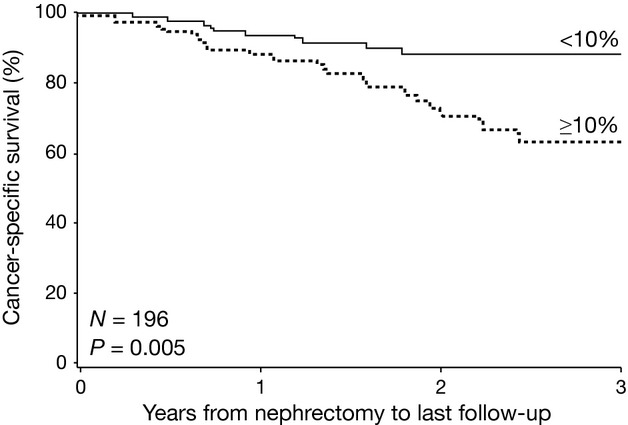
Increased PD-L1 expression (≥10% vs. <10%) diminishes survival in RCC. From Thompson et al. 34. Copyright 2012 National Academy of Sciences, U.S.A. Reprinted with permission.

Expression of PD-1 by tumor-infiltrating lymphocytes (TILs) is associated with impaired effector function (cytokine production and cytotoxic efficacy against tumor cells) and/or poor outcome in several tumor types 29–32. Moreover, a variety of tumors, including RCC, MEL, as well as stomach, breast, ovarian, pancreatic, and lung cancers, have been shown to express PD-L1, potentially contributing to immune suppression and evasion 33. Of note, PD-L1 expression on tumor cells has been shown to correlate with poor prognosis in patients with RCC (Fig. 2) 34, MEL, and breast, pancreatic, stomach, bladder, lung, liver, and ovarian cancers (Table 2) 33,35–38. However, a recent report has shown that not all tumor PD-L1 expression confers a worse prognosis 27. In this study, patients with PD-L1^+^ MEL survived significantly longer than patients with PD-L1^−^ MEL, spurring investigators to hypothesize that TILs may actually prompt their own inhibition by secreting cytokines (such as IFN-γ) that drive tumor PD-L1 expression. However, because of the significant number of patients who received subsequent immune-based therapies, care must be taken interpreting these data. It is, however, possible that induction of the PD-L1/PD-1 pathway represents a mechanism of adaptive resistance exerted by tumors infiltrated by effector T cells, and is therefore a marker of an ongoing antitumor immune response. Similarly, PD-L2 has been observed to be upregulated in a subset of human tumors and has occasionally been linked to poor outcome 39. Consequently, therapies that block the PD-1 pathway may unleash antitumor immunity and be particularly beneficial to patients with PD-L^+^ tumors.

**Table:** PD-L1 expression and clinical outcomes in cancer

Disease	PD-L1 expression associations
RCC	Poor prognosis 30
NSCLC	Decreased survival 35
MEL	Increased 24 or decreased 32 survival
Breast	Tumor size, stage, and HER2 expression 30
Gastric	Tumor size, metastasis, and poor survival 30
Ovarian	Poor prognosis 30
Pancreatic	Decreased TILs and poor prognosis 30
HCC (HBV related)	Poor postcryoablation prognosis^1^,^33^
HCC	Tumor aggressiveness and recurrence after resection 34

HBV, hepatitis B virus; HCC, hepatocellular carcinoma; MEL, melanoma; NSCLC, non–small cell lung cancer; RCC, renal cell cancer.

Associated with PD-L1 expression on circulating leukocytes.

Ahmadzadeh and colleagues showed that PD-1 expression on TILs in MEL lesions was significantly higher than expression on T cells isolated from the peripheral blood (PB) or noncancerous tissue from the same patients or healthy donors 31. In this study, PD-1^+^ TILs had impaired effector function, as measured by IFN-γ secretion, indicating that PD-1 expression on TILs limits their capacity to mount an effective immune response. Patients with MEL also have higher levels of PD-1 expression on TILs than on PB lymphocytes 40. In addition, blockade of the PD-1/PD-L1 pathway increased IFN-γ secretion by T cells in response to stimulation by antigen-loaded DCs. Overall, these findings suggest that inhibition of the PD-1/PD-L1 pathway can restore effector functions of exhausted T cells, which may translate into improved antitumor immune responses. Thus, the PD-1/PD-L1 pathway represents a logical target for cancer immunotherapy.

## Preclinical Studies of PD-1 Blockade

Blockade of either PD-1 or its ligands has shown consistent immune-potentiating effects in a range of preclinical models. Antibodies against PD-1 or PD-L1 can enhance or restore T-cell effector function, including cytolytic activity against tumor cells 40–42. Moreover, PD-L1 blockade promoted infiltration of tumor-reactive CD8+ T cells into established tumors in a mouse model of pancreatic cancer 43, while PD-L2 blockade decreased the numbers of tumor-infiltrating regulatory T cells 44. PD-1 blockade inhibited the metastatic spread of MEL and colon cancer cells in mice 45. Experiments in mice lacking PD-1 showed that hematogenous tumor spread was inhibited via several T-cell potentiating mechanisms, including enhanced induction of effector T cells in the spleen, augmented homing of these cells to tumor sites, and improved T-cell proliferation and cytotoxicity 45. Several preclinical studies have shown that PD-1 or PD-L1 blockade improves the immune response to, and/or efficacy of, other immunotherapies, including anti–CTLA-4 mAbs 41–43,46. PD-L1 inhibition was also synergistic with chemotherapy in a mouse model of pancreatic cancer 43. These observations have important implications for the development of potential combination treatment strategies for patients with cancer.

## Clinical Studies Investigating PD-1/PD-L1 Blockade

Because of the varied negative signals resulting from interactions between PD-1/PD-L1/PD-L2 and PD-L1/CD80, there are opportunities to target this checkpoint pathway from two directions: PD-1 blockade inhibits negative signaling induced by PD-L1 and PD-L2 ligation, whereas PD-L1 blockade inhibits negative signaling via PD-1 and CD80. MAbs that target these interactions are being evaluated in clinical studies and hold promise as an important immunotherapeutic approach in various malignancies. Data from clinical trials targeting the interactions between PD-1 and its ligands have recently become available.

## PD-1 Blockade with Anti–PD-1 Antibodies

### Nivolumab

The first phase 1 trial of varying doses (0.3–10 mg/kg) of nivolumab, a fully human IgG4 anti–PD-1-blocking antibody, in 39 patients with advanced MEL, CRC, CRPC, NSCLC, or RCC was reported in 2010 19. One complete response (CR) (CRC 3 mg/kg) (21+ months at last follow-up) and two partial responses (PRs) (RCC and MEL [both 10 mg/kg]) were observed. No maximum tolerated dose (MTD) was identified. Most adverse events (AEs) reported for nivolumab were immune-related, consistent with the mechanism of action of anti–PD-1 antibodies. Flow cytometric analysis showed sustained occupancy of the majority of PD-1 molecules on circulating T cells isolated from patient plasma samples for ≥3 months following a single dose of nivolumab. In an expansion cohort at the 10-mg/kg dose level, responses were seen in patients with MEL and RCC without increased toxicity. One patient with RCC who had received several prior therapies achieved a PR that lasted 16+ months despite receiving only three nivolumab infusions. Additionally, two patients (NSCLC, MEL) achieved significant regression of metastatic lesions that did not meet PR criteria 19. Follow-up of three patients from this study has demonstrated long-term responses after discontinuation of therapy 47.

**Figure 3:**
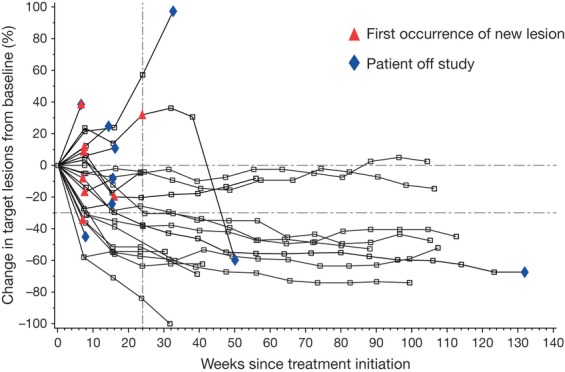
Durable responses in a cohort of patients with MEL treated with 1 mg/kg nivolumab. From Topalian et al. 11. Copyright 2012 Massachusetts Medical Society. Reprinted with permission.

The activity seen in the initial study prompted an exploration of a biweekly schedule of nivolumab in a separate phase 1 trial 11,48. Interim data from 304 patients with RCC, MEL, CRPC, NSCLC, or CRC who received 0.1 mg/kg to 10 mg/kg nivolumab every 2 weeks in 8-week cycles have recently been reported. Patients received treatment (≤12 cycles) until they experienced CR or progressive disease. Of 294 response-evaluable patients, there were objective responses (ORs) in 20/122 patients with NSCLC, 33/106 patients with MEL, and 10/34 patients with RCC. No ORs were noted in patients with CRPC or CRC. At last follow-up, 28 patients to date have had responses lasting ≥1 year. Stable disease (SD) lasting 24+ weeks was observed in an additional six evaluable patients with MEL, 11 with NSCLC, and nine with RCC. Additional patients with each disease experienced tumor responses to nivolumab according to immune-related response criteria; one pattern is typified by an initial increase and then decrease in tumor size (Fig. 3). Antitumor activity was observed at 1- to 10-mg/kg dose levels; some patients appeared to continue to exhibit tumor response beyond 96 weeks, the protocol-defined treatment stoppage point. The most common treatment-related AEs included fatigue, rash, nausea, diarrhea, decreased appetite, and pruritus. Grade 3/4 treatment-related AEs occurred in 15% of patients, and there were three deaths, all attributed to pulmonary toxicity. Drug-related AEs of special interest (AEOSIs, AEs with potentially immune-related etiology) included vitiligo, pneumonitis, hepatitis, colitis, thyroiditis, and hypophysitis. Should this preliminary efficacy and relatively favorable toxicity profile be confirmed in future trials, PD-1 pathway blockade may be an approach that can produce a durable benefit with fewer toxicities than with other immunotherapies (e.g., HD IL-2 or ipilimumab). Nivolumab is currently being evaluated in several ongoing clinical trials of patients with RCC, MEL, and various other malignancies (http://clinicaltrials.gov).

### Pidilizumab

The humanized, anti–PD-1 mAb pidilizumab was evaluated in a phase 1 clinical trial of patients with advanced hematologic malignancies 13. Seventeen patients received escalating doses of pidilizumab ranging from 0.2 to 6 mg/kg. Treatment with pidilizumab generally was well tolerated, and no MTD was defined. Only one patient reported toxicity, specifically weakness, and flushing, which were possibly treatment related. Clinical benefit was observed in 33% of patients. An apparent response to treatment was observed in six patients, including one CR, four SD, and one mixed response; the CR was maintained for ≥68 weeks. Sustained elevation in PB CD4^+^ lymphocyte numbers was observed up to 21 days following treatment with pidilizumab 13. The activity of pidilizumab in solid tumors is now being explored (http://clinicaltrials.gov).

### Lambrolizumab

Patnaik and colleagues reported the results of a small, open-label dose-escalation study examining lambrolizumab, a humanized IgG4 mAb against PD-1 12. Cohorts of three to six patients with various advanced solid tumors were treated with 1–10 mg/kg initially and then additional doses every 2 weeks. No MTD was identified, and there were no grade ≥3 drug-related AEs; one patient developed pneumonitis, which was treated with corticosteroids. Two patients with MEL treated for 6+ months had PRs, and there was an unconfirmed PR in a patient with squamous NSCLC. In the MEL expansion cohort treated with 10 mg/kg, one unconfirmed CR, three unconfirmed PRs, and five grade 3/4 AEs were reported (NCT01295827) 12. Additional interim data on 85 evaluable patients were recently presented at the Society for Melanoma Research Congress 49, Forty-three patients (51%) experienced ORs, including eight CRs. 27 patients had previously been treated with ipilimumab and 11 of these (41%) had ORs. Seven treatment-related grade 3/4 AEs with potentially immune-mediated causes were reported. There were three instances of grade 1/2 pneumonitis; one patient died of myocardial infarction while being treated for pneumonitis/pneumonia. Common AEs included arthralgia, cough, diarrhea, fatigue, fever, nausea, pruritus, and rash.

### Patterns of response to PD-1 blockade

While a subset of melanoma and lung cancer patients treated with PD-1-pathway–targeted agents have experienced encouraging ORs, fewer have experienced SD. It is possible that the response to PD-1 pathway blockade is “all or nothing.” In comparison, while only a small percentage of patients treated with ipilimumab achieve ORs, this agent improved median OS in phase 3 trials, suggesting a clinical benefit in patients who did not meet response criteria 9,50. It is possible that PD-1-pathway–directed agents may impart a survival benefit only to those patients on the “tail” of the survival curve. Whether these agents will improve median OS in unselected patients will be determined by ongoing, randomized phase 3 trials.

It should be noted that select responding patients who discontinued PD-1 antibody therapy subsequently demonstrated disease progression, suggesting that the optimal duration of anti–PD-1 agent dosing may vary from patient to patient. However, some patients who progressed after discontinuation of anti–PD-1 therapy have demonstrated durable responses after retreatment 47. The optimal duration of PD-1-pathway–targeted agent treatment is still being determined; if shorter treatment durations are proven efficacious, this approach would be more cost-effective. The ultimate benefit of immunotherapeutics (e.g., IL-2, ipilimumab) is their ability to produce remissions that are durable when therapy is discontinued. Although this has been observed in select patients treated with anti–PD-1 agents, the percentage of patients who achieve durable remissions remains to be determined. Nonetheless, the potential benefits for patients who experience “treatment-free survival” include decreased treatment-associated toxicity, improved quality of life, and decreased cost to the healthcare system. While this endpoint is not currently standard for approving therapies, its value to the patient merits consideration in future studies.

## PD-1 Blockade with PD-1 Ligand-targeted Agents

### BMS-936559

Interim data from a phase 1 trial (NCT00729664) of BMS-936559, an anti–PD-L1 mAb, have recently been disclosed 14. As of 24 February 2012, 207 patients (with NSCLC, MEL, CRC, RCC, and ovarian, pancreatic, or breast cancer) had received BMS-936559 (at escalating doses ranging from 0.3 to 10 mg/kg) every 2 weeks in 6-week cycles. This continued for 16 cycles or until progressive disease or CR; the MTD was not identified. In the evaluable population, ORs were observed in patients with MEL (9/52), RCC (2/17), NSCLC (5/49), and ovarian cancer (1/17). In the 16 patients with ≥1 year of follow-up, responses in half lasted for ≥1 year. SD (≥24 weeks) was observed in 14 patients with MEL, six patients with NSCLC, three patients with ovarian cancer, and seven patients with RCC. Most AEs were low grade; the most common treatment-related AEs included fatigue, diarrhea, infusion reactions, rash, arthralgia, headache, nausea, and pruritus. Grade 3/4 treatment-related toxicities occurred in 9% of patients. Thirty-nine percent of patients experienced AEOSIs, including rash, hepatitis, hypothyroidism, and one case each of diabetes mellitus, endophthalmitis, sarcoidosis, and myasthenia gravis. The data collected to date demonstrate that BMS-936559 has a relatively good safety profile and can induce durable responses in patients with MEL, NSCLC, and RCC.

### Other agents

Genentech and Roche are evaluating a mAb targeting PD-L1 (RG7446/MPDL3280A), and Amplimmune and GlaxoSmithKline have partnered in the development of a PD-L2/IgG1 fusion protein (AMP-224) that blocks PD-1 signaling. More recently, MedImmune announced that it would pursue development of the anti–PD-L1 mAb MEDI4736 51. All three of these compounds are currently in phase 1 testing (http://clinicaltrials.gov).

### Targeting PD-1 versus PD-L1

Without a randomized, head-to-head trial of these drugs, no conclusive statements can be made regarding the comparative safety and activity of agents targeting PD-1 versus those targeting PD-L1. It appears, however, that targeting only PD-L1 may be accompanied by less toxicity, but also may be less effective than targeting PD-1 and thus blocking signaling via both PD-L1 and PD-L2.

### Differences from anti–CTLA-4 antibodies

The anti–PD-1 data we currently have are from phase 1 trials, and no randomized, head-to-head studies of anti–PD-1 and anti–CTLA-4 antibodies have been completed. However, the existence of these agents in the same immunotherapeutic category will no doubt prompt comparisons. Both PD-1 and CTLA-4 are checkpoint molecules; however, they participate in different phases of the immune response. Murine experiments suggest that CTLA-4 is centrally involved in the development of tolerance and the prevention of autoimmunity, while the role of PD-1 is to prevent bystander tissue damage at sites of chronic infection and inflammation 52–54. Additionally, given the body of data on PD-L1 and PD-1 as potential prognostic and predictive biomarkers, it seems that this pathway may be more important in the tumor microenvironment. Preclinical data 46 suggest that the combination of inhibitors of PD-1 and CTLA-4 may present a promising approach, which is currently being explored in the clinical setting [NCT01454102, NCT01024231].

## Future Directions

### Predictive biomarker development

The correlation between tumor PD-L1 expression and prognosis supports the hypothesis that this molecule may serve as a predictive biomarker. Although immune infiltration of antitumor lymphocytes leading to PD-L1 upregulation might establish a scenario predictive of benefit for any effective immunotherapy, this is particularly true of agents targeting the PD-1 pathway. As mentioned previously, expression of PD-1 by TILs is associated with poor prognosis in multiple cancers 29,32. In patients with RCC, the presence of PD-1^+^ TILs was associated with more aggressive tumors and shortened survival 29. In addition, patients with PD-1^+^ TILs were more likely than patients without PD-1^+^ TILs to have larger tumors, tumors of higher nuclear grade, advanced tumor node-metastasis stage, coagulative tumor necrosis, and sarcomatoid differentiation. In RCC and other cancers, tumor expression of PD-L1 is also associated with poor prognosis and more aggressive disease 33,34. However, the significance of PD-L1 expression in the context of active immunotherapy, particularly PD-1/PD-L1 pathway blockade, is less clear. Given that tumor PD-L1 expression is induced by T-cell expression of IFN-γ, it may signify an effort by the tumor to evade an ongoing immune response. Therefore, blockade of the PD-1/PD-L1 pathway with an anti–PD-1 or anti–PD-L1 antibody may enable the unmasking of antitumor immunity patients with PD-L1^+^ tumors. Preliminary data suggesting that tumor PD-L1 expression could be a predictive biomarker of response to nivolumab 19 were later supported by an observation that 0/17 nivolumab-treated patients with PD-L1^−^ tumors experienced ORs, whereas 9/25 patients with PD-L1^+^ tumors (36%) achieved ORs (*P *=* *0.006). If these data are confirmed, PD-L1 positivity may be a way to identify and enrich the population of patients who benefit from PD-1 pathway–targeted therapy. Further evaluation of PD-L1 as a potential predictive marker is warranted.

### Opportunities for combination therapy

Even at this stage of PD-1–targeted agent development, it is important to consider opportunities for combination therapy. Given the observation that multiple factors can induce PD-L1 expression, it may be feasible to treat patients with these molecules (e.g., IFN-α, -β, and -γ 16; cisplatin 55) to induce PD-L1 expression in the tumor microenvironment and expand the population of patients who might benefit from treatment with PD-1-pathway–directed agents. In addition, tumors can evade detection and destruction by manipulating the immune system: downregulating costimulatory molecules on tumor cells, increasing expression of immunosuppressive molecules, and dysregulating T cells and APCs. Therefore, the implementation of rational, multiagent treatments that target distinct pathways may circumvent these tumor survival strategies.

#### Dual checkpoint blockade

The combination of CTLA-4 and PD-1 blockade may also enable more persistent immune activation while avoiding the major toxicities associated with HD IL-2. Hypothetically, combining two agents that target T-cell activation at different stages of the immune response will be a more potent anticancer treatment than therapy with each agent alone. Ipilimumab removes a physiological brake on T cells during activation, whereas anti–PD-1 removes a brake on activation during T-cell effector function. This combination may also overcome resistance to CTLA-4 blockade mediated by tumor PD-L1 expression or resistance to PD-1 blockade mediated by T-cell downregulation through the coexpression of CTLA-4. A phase 1 trial of nivolumab and ipilimumab in patients with MEL is ongoing (NCT01024231). Another potential checkpoint combination therapy might include blockade of PD-1 and LAG-3, a molecule also involved in the regulation of T-cell activation. Combination therapy in mice has shown impressive activity 56.

#### Targeted therapy combinations

The combination of agents that block immune downregulation with genetic or cell-targeted therapies may prove particularly effective in select patients. Following this line of thought, pidilizumab is being tested in combination with rituximab, an anti-CD20 antibody, in a phase 2 trial of patients with relapsed follicular lymphoma (NCT00904722). The combination of PD-1–targeted therapy with agents effective against genetic mutations may also demonstrate activity. Trials examining vemurafenib, an inhibitor of B-raf enzymes with the V600E mutation, and other BRAF and epidermal growth factor receptor–targeted agents, in combination with PD-1 pathway inhibitors are under way (NCT01454102, NCT01656642). Various chemotherapies are also being evaluated in combination with anti–PD-1 agents (NCT00890305, NCT01454102).

#### T-cell stimulating agent combinations

IL-2 is a cytokine that supports T-cell survival and proliferation. The combination of HD IL-2 and ipilimumab has demonstrated manageable toxicity and impressive efficacy (CR rate of 17%) in patients with advanced MEL 57. Thus, it is conceivable that the combination of HD IL-2, to induce T-cell expansion, and PD-1 blockade, to eliminate tumor-induced immune suppression, might prove equally or more efficacious in select patients.

#### Vaccine combinations

The PD-1/PD-L1 pathway may play an important role in blunting immune response to tumor vaccines. Study findings suggest that stimulation with dendritic/tumor fusion cells increases T-cell expression of PD-1, which may blunt the host response to vaccination 58. In ex vivo studies, stimulation of T cells with a DC/tumor fusion vaccine in the presence of PD-1 blockade resulted in increased cytokine production, decreased Tregs, and enhanced tumor killing 58. As such, combining DC-based tumor vaccines with PD-1 blockade may be an effective means of enhancing immunologic and clinical response to vaccination. Interestingly, two trials evaluating the efficacy of nivolumab in combination with multipeptide melanoma vaccines are ongoing (NCT01176474, NCT01176461). Thus far, confirmed PRs have been reported in patients with unresectable melanoma at each of the treating dose levels (two at 1 mg/kg, four at 3 mg/kg, and one at 10 mg/kg), and the most common drug-related AEs are low-grade injection site reactions and nausea/vomiting 59. Pidilizumab is being tested in combination with DC fusion vaccines in patients with multiple myeloma after stem cell transplantation (NCT01067287), patients with RCC (NCT01441765), and patients with acute myeloid leukemia (NCT01096602).

#### VEGFR tyrosine kinase inhibitor (TKI) combinations

The anti–CTLA-4 mAb tremelimumab administered with the vascular endothelial growth factor receptor (VEGFR) TKI sunitinib produced PRs in 9/21 evaluable patients with RCC, but was associated with acute renal toxicity, which the authors proposed might be immune related 60. It is conceivable that combinations involving VEGFR TKIs with PD-1/PD-L1 pathway blockade may be better tolerated. Thus, an ongoing trial is assessing the safety and tolerability of nivolumab when administered with sunitinib or pazopanib in patients with RCC (NCT01472081) 61.

#### VEGF-targeted agent combinations

There is evidence that VEGF decreases DC function 62, and by extension, antigen presentation and T-cell activation. Concurrently restoring DC and T-cell functionality may improve the antitumor T-cell response and lead to improved clinical activity. As proof-of-concept, Hodi and colleagues have observed durable PRs in patients with MEL treated with a combination of ipilimumab and the anti–VEGF-A antibody bevacizumab 63. Trials evaluating the combination of nivolumab (NCT01454102) or ipilimumab (NCT00790010) with bevacizumab are ongoing (http://clinicaltrials.gov).

Various benefits and drawbacks accompany the use of broad spectrum versus more specific VEGF inhibitors. Broad-spectrum agents are indeed pleiotropic and, among various “off-target effects” can impact immune system function. For instance, murine experiments have shown that sunitinib can decrease Treg populations 64; other TKIs are associated with immune system-potentiating effects 65. If the pleiotropic effects of a given VEGFR TKI have a net positive effect on the immune system and are not overwhelmingly detrimental to other physiological processes, it may indeed be worth exploring in combination with anti–PD-1-pathway–directed agents. In contrast, VEGF-targeted agents like bevacizumab and VEGF-trap are more specific in their inhibition, but are not typically accompanied by immune-potentiating effects. As high levels of VEGF are associated with impeded dendritic cell function, lowering environmental VEGF concentrations might benefit this aspect of the antitumor immune response. In recent phase 3 studies, bevacizumab administered in combination with IFNα was more effective than IFNα alone 66, suggesting that combination therapy of PD-1–directed agents and VEGF-binding antibodies merits future study.

## Conclusions

Although first-generation immunotherapies were of limited efficacy, they provided proof-of-concept that, in some patients, the immune balance in the tumor could be shifted in favor of tumor elimination. Expanding knowledge and understanding of the immune system's role in cancer has revealed multiple mechanisms by which tumors evade immune destruction. In particular, negative regulatory pathways involved in the T-cell-mediated response, including interaction of PD-1 and PD-L1, are being investigated to determine their role in tumor development. Various solid tumors express PD-L1, often associated with poor prognosis, whereas TILs from these patients express PD-1 and have impaired antitumor functionality. Preclinical proof-of-concept for PD-1/PD-L1 blockade has been demonstrated by many studies: improvements in various antitumor T-cell functions have been observed. Several PD-1 and PD-L1-blocking mAbs have now completed early clinical development with encouraging activity and safety. The potential for these agents in the treatment of patients with advanced cancers, including their incorporation into combination regimens (e.g., PD-1- plus CTLA-4-blocking antibodies), is significant, and further data are eagerly awaited.
